# Crucial Role of Stromal Interaction Molecule-Activated TRPC-ORAI Channels in Vascular Remodeling and Pulmonary Hypertension Induced by Intermittent Hypoxia

**DOI:** 10.3389/fphys.2022.841828

**Published:** 2022-03-17

**Authors:** Sebastián Castillo-Galán, Bárbara Riquelme, Rodrigo Iturriaga

**Affiliations:** ^1^Laboratorio de Neurobiología, Facultad de Ciencias Biológicas, Pontificia Universidad Católica de Chile, Santiago, Chile; ^2^Centro de Excelencia en Biomedicina de Magallanes (CEBIMA), Universidad de Magallanes, Punta Arenas, Chile

**Keywords:** 2-APB, STIM-activated TRPC-ORAI channels, chronic intermittent hypoxia, pulmonary hypertension, pulmonary vascular remodeling, hypoxia, obstructive sleep apnea

## Abstract

Obstructive sleep apnea (OSA), a sleep breathing disorder featured by chronic intermittent hypoxia (CIH), is associate with pulmonary hypertension. Rats exposed to CIH develop lung vascular remodeling and pulmonary hypertension, which paralleled the upregulation of stromal interaction molecule (STIM)-activated TRPC-ORAI Ca^2+^ channels (STOC) in the lung, suggesting that STOC participate in the pulmonary vascular alterations. Accordingly, to evaluate the role played by STOC in pulmonary hypertension we studied whether the STOC blocker 2-aminoethoxydiphenyl borate (2-APB) may prevent the vascular remodeling and the pulmonary hypertension induced by CIH in a rat model of OSA. We assessed the effects of 2-APB on right ventricular systolic pressure (RVSP), pulmonary vascular remodeling, α-actin and proliferation marker Ki-67 levels in pulmonary arterial smooth muscle cells (PASMC), mRNA levels of STOC subunits, and systemic and pulmonary oxidative stress (TBARS) in male Sprague-Dawley (200 g) rats exposed to CIH (5% O_2_, 12 times/h for 8h) for 28 days. At 14 days of CIH, osmotic pumps containing 2-APB (10 mg/kg/day) or its vehicle were implanted and rats were kept for 2 more weeks in CIH. Exposure to CIH for 28 days raised RVSP > 35 mm Hg, increased the medial layer thickness and the levels of α-actin and Ki-67 in PASMC, and increased the gene expression of TRPC1, TRPC4, TRPC6 and ORAI1 subunits. Treatment with 2-APB prevented the raise in RVSP and the increment of the medial layer thickness, as well as the increased levels of α-actin and Ki-67 in PASMC, and the increased gene expression of STOC subunits. In addition, 2-APB did not reduced the lung and systemic oxidative stress, suggesting that the effects of 2-APB on vascular remodeling and pulmonary hypertension are independent on the reduction of the oxidative stress. Thus, our results supported that STIM-activated TRPC-ORAI Ca^2+^ channels contributes to the lung vascular remodeling and pulmonary hypertension induced by CIH.

## Introduction

Obstructive sleep apnea (OSA) characterized by cyclic episodes of intermittent hypoxia is a health problem that affect a large worldwide adult population (Somers et al., 2008; Dempsey et al., 2010; Floras, 2018). Obstructive sleep apnea is associated with somnolence, sleep fragmentation and cognitive dysfunction, but it is also considered as an independent risk factor for systemic diurnal hypertension (Somers et al., 2008; Dempsey et al., 2010; Iturriaga et al., 2021). Furthermore, OSA is associated with mild pulmonary hypertension with an incidence ranging from 20-50% (Sajkov and McEvoy, 2009; Floras, 2018). Chronic intermittent hypoxia (CIH) is the main factor for develop systemic hypertension in OSA patients (Somers et al., 2008; Dempsey et al., 2010) and animal exposed to CIH (Del Rio et al., 2010, 2014; Iturriaga et al., 2014, 2021; Iturriaga, 2018; Harki et al., 2021). In addition, CIH produces vascular pulmonary remodeling and increases right ventricular systolic pressure (RVSP) in rodents (Nisbet et al., 2009; Jin et al., 2014, 2016; Iturriaga and Castillo-Galán, 2019; Castillo-Galán et al., 2020). Recently, we found that CIH progressively increased the right ventricular systolic pressure (RVSP) over 35 mm Hg in the rat, enhanced the vasoconstrictor response of small pulmonary arteries to KCl and endothelin-1 (ET-1), increased the medial layer thickness, and increased the right-ventricular wall thickness and the right-ventricular end systolic volume related to the control rats (Castillo-Galán et al., 2020). However, even though the link between OSA and pulmonary hypertension is well recognized, the underlying pathogenic mechanisms are not completely known. The pathological pulmonary vascular alterations induced by CIH has been attributed to Ca^2+–^dependent enhanced contractility and proliferation of pulmonary arterial smooth muscle cells (PASMC) induced by the activation of hypoxic inducible factors (HIFs) and oxidative stress (Nisbet et al., 2009: Jin et al., 2014, 2016; Iturriaga and Castillo-Galán, 2019). Thus, it is likely that the activation of HIFs and reactive oxygen species (ROS)-dependent signaling pathways may increase intracellular PASMC calcium levels contributing to the development of the CIH-induced enhanced vasoconstriction, cell proliferation and pulmonary hypertension (Waypa et al., 2002; Sylvester et al., 2012).

Among channels and transporters involved in Ca^2+^ intracellular homeostasis, it has been proposed that store operated calcium entry contributes to enhance the PASMC contractility in sustained hypoxia (Kuhr et al., 2012; Wang et al., 2012, 2017; Reyes et al., 2018). The binding of endothelin-1 (ET-1) or serotonin (5-HT) to their receptors coupled to G-protein evokes the release of the second messenger IP_3_, which in turn activates intracellular calcium channels (IP3R) depleting the Ca^2+^ stores of the sarcoplasmic reticulum (Zhang et al., 2003; Wang et al., 2012). STOC are activated by a depletion of Ca^2+^ stores in the sarcoplasmic reticulum, which allow the entry of calcium from the extracellular space. A growing body of evidence supports the contribution of STOC in the contraction of pulmonary arteries in response to acute and sustained hypoxia (Weigand et al., 2005; Lu et al., 2008, 2009; Ng et al., 2008; Guibert et al., 2011). Furthermore, STOC-mediated calcium entry participates in pathological pulmonary vascular remodeling process, such as hypercontractility, proliferation and differentiation of PASMC (Golovina et al., 2001; Hou et al., 2013; Reyes et al., 2018). From a structural point of view, STOC are composed by homo or heterotetramer pore-forming subunits from the TRPC family (TRPC 1 to 7), or to the ORAI family (ORAI 1 to 3) (Reyes et al., 2018). On the other hand, an auxiliary subunit of the family of stromal interaction molecules [stromal interaction molecule (STIM) 1 and 2] acts as a calcium sensor activating pore-forming subunits in response to calcium depletion from sarcoplasmic reticulum deposits (Fuchs et al., 2010; Johnstone et al., 2010; Wray and Burdyga, 2010; Yang et al., 2010). Indeed, a growing body of evidence suggests that STIM-activated TRPC-ORAI Ca^2+^ channels (STOC) play a key role in PASMC remodeling and hypercontractility, which led to pulmonary hypertension in animals exposed to sustained hypoxia (Weigand et al., 2005; Ng et al., 2008; Castillo-Galán et al., 2016, 2022)

Recently, we found that CIH produced the upregulation of mRNA and protein levels of TRPC1, TRPC4, TRPC6, ORAI1, and STIM1 subunits in the rat lung tissue, which paralleled the vascular remodeling and pulmonary hypertension (Castillo-Galán et al., 2020), suggesting that STOC participate in the vascular alterations induced by CIH. Accordingly, to assess the contribution of STOC on the CIH-induced pulmonary vascular alterations, we study the effects of the STOC non-selective inhibitor 2-aminoethyldiphenylborinate (2-APB) (Bootman et al., 2002; Smith et al., 2015; Castillo-Galán et al., 2016, 2022; Jain et al., 2021) on the development of pulmonary hypertension and vascular remodeling induced by CIH in a well stablished preclinical model of OSA (Del Rio et al., 2010, 2012, 2014; Iturriaga et al., 2014, Iturriaga and Castillo-Galán, 2019). We also determined the effects of 2-APB on systemic and lung oxidative stress induced by CIH, because 2-APB ameliorates the oxidative stress in a heart ischemia-reperfusion model in mice (Morihara et al., 2017) and rat (Shen et al., 2021).

## Materials and Methods

### Animal Model and Intermittent Chronic Hypoxia Protocols

The experimental protocols were approved by the Scientific Ethical Committee for the Animal and Environment Care from the Pontificia Universidad Católica de Chile, Santiago, Chile (ID:180803006), and were performed according to the National Institutes of Health Guide (NIH, United States) for the care and use of animals. The experiments were performed in 19 male Sprague-Dawley rats (∼ 200 g) from the Center from Innovation in Biomedical Experimental Models (CIBEM) of the Pontificia Universidad Católica de Chile. Rats were fed with a standard diet and access to food and water *ad libitum* and the room temperature was maintained between 23 and 25°C. Rats were exposed to CIH (5% O_2_ for 20 s, followed by 290 s of normoxia, 12 time for 8 h/day, from 9:00 AM. to 5:00 PM. for 28 days. The O_2_ levels in the chambers were regulated with a computerized system, which open and close solenoid valves that control the entry of N_2_ and its removal by a fan (Del Rio et al., 2010, 2012, Castillo-Galán et al., 2020).

### 2-Aminoethyl Diphenylborinate Administration

Rats were randomized divided into 3 groups (*n* = 6–7). At 14 days of CIH, rats were anesthetized with isoflurane 2% in 100% O_2_, and mini osmotic-pumps (2ML4, Alzet Scientific Products, MD, United States) were implanted subcutaneously in the back. Pumps were loaded with 2-APB (Sigma-Aldrich, United States) to produce a continuous release of 10 mg/kg/day or its vehicle (Dimethyl sulfoxide: ETOH: Saline 1: 5: 4). After surgery, both groups were exposed to CIH for 14 more days, and the results were compared with the control group of rats exposed to normoxia.

### Right Ventricular Systolic Pressure Measurement

At the end of 28 days of CIH, rats were anesthetized with urethane/α-chloralose (40/800 mg/kg, i.p.), tracheostomized and artificially ventilated with a RoVent Jr ventilator (Kent Scientific, Torrington, CT, United States). A thoracotomy was performed to expose the heart, and the right ventricular systolic pressure (RVSP) was recorded with a heparinized catheter inserted into the right ventricle connected to a Statham P23 transducer (Hato Rey, Puerto Rico, United States). The heart rate was derived from the pressure signal recorded with a PowerLab 16 channels acquisition system (ADInstruments, Castle Hill, Australia) (Castillo-Galán et al., 2020).

### mRNA Extraction and RT-qPCR

Frozen left lung samples (∼100 mg) were homogenized in TRIzol (Thermo Fisher Scientific, Waltham, MA, United States). The RNA was resuspended in nuclease-free water (20 μl) to later determination of its concentration and purity by spectrophotometry, being the 260/280 ratio of all samples in the range of 1.9–2.0. The RNA was stored at −80°C until later use. Synthesis to cDNA was performed using the cDNA Synthesis Kit (ABM, Richmond, Canada). The gene expression of TRPC1, TRPC4, TRPC6, ORAI1, ORAI2 and 18S was determined by real-time PCR (qPCR) in a StepOne Plus equipment (Applied Biosystems, Foster City, CA, United States), using a KicqStart kit (Sigma-Aldrich, United States) with the primers sequence used by Castillo-Galán et al. (2020). Relative gene expression was calculated with the 2^–ΔΔCt^ method using 18S gene.

### Malondialdehyde Concentration

The malondialdehyde (MDA) concentration was measured using a specific enzyme immunoassay thiobarbituric acid reactive substances (TBARS) kit (Cayman Chemical, Ann Arbor, MI, United States) Briefly, 25 mg of fresh lung tissue were homogenized in RIPA Buffer Concentrate (Cayman Chemical, Ann Arbor, MI United States) and centrifuged at 1600 x *g* for 10 min at 4°C. The supernatant were collected for analysis. Blood samples were collected from the left ventricle using a syringe with heparin. Blood samples were centrifuged at 1000 x *g* for 10 min at 4°C and the fresh plasma (supernatant) were use for analysis.

### Vascular Remodeling and Immunohistochemistry

Samples of ∼1 cm of the external portion of the medium lobe from the left lung containing distal pulmonary arterioles were collected. The lung tissue was fixed by immersion in Paraformaldehyde 4%, included in paraffin blocks, and cut in 3–5 μm slides. For the morphological analysis, the slides were deparaffinized, dehydrated and stained with Van Gieson. Using a trinocular microscope (Olympus BX41, Japan) with a digital camera (Jenoptik Progres C3), 10–15 images of 150–300 μm internal diameter pulmonary arteries were acquired per lung from each animal. The area of the muscle layer and the artery lumen were measured using the Image Pro6.3 Software (Sigma-imaging, United Kingdom). The percentage of muscle layer was determined using the following formula: (muscle area - lumen area)/muscle area * 100, according to Castillo-Galán et al. (2020).

Immunohistochemical detection of α-actin and Ki-67 (α-actin-r and KI-67-ir) was performed with the smooth muscle α-actin antibody (Clone RM253, Sigma-Aldrich, Misuri, United States, dilution: 1:400) and the Ki-67 antibody (Clone MIB-1, Dako, Glostrup, Denmark, dilution 1:100). For all procedures, the antigen retrieval was performed at 100°C with citrate buffer at pH 6.0. Primary antibody incubations were performed overnight at 4°C in a wet chamber. The labeling was developed using a HRP-diaminobenzidine kit (Envision, Dako, CA, United States). Lung tissue imagen were digitally acquired at 40×. The α-actin-ir in the muscle layer of small pulmonary arteries was measured as pixel count per area. The percentage of KI-67-ir positive nuclei in the medial layer of PMASC was calculated as the percentage of positive KI-67 nuclei (Castillo-Galán et al., 2016).

### Vascular Luminal Surface and Vascular Density

In histological sections of lung stained with Van Gieson, 6 images were captured with a magnification of 10×. Subsequently, the vascular luminal surface (luminal area/total area) and the vascular density (number of arteries/total area) were determined in arteries of 150–300 μm of internal diameter.

### Statistical Analysis

Data were expressed as mean ± standard standard desviation (SD). One-way or two way ANOVA followed by a Newman-Keuls post-test was used for the analysis. Results were analyzed and plotted in GraphPad Prism 8 software (GraphPad Software Inc., San Diego, CA, United States). For all comparisons, differences were considered statistically significant when *P* < 0.05.

## Results

### Animal Weight Gain

All groups showed a similar gain of weight during the experiments (*P* < 0.001, two-way ANOVA, between initial and final weight in each group). However, we did not find differences in the initial and final weight between the groups (*P* = 0.063, two way-ANOVA) (Table 1).

**TABLE 1:** Initial and final weights of the rats during the experiments.

Group	Initial weight (g)	Final weight (g)
Control	207.7 ± 14.5	290.0 ± 18.8 ##
Vehicle + 28 d CIH	199.5 ± 10.5	302.8 ± 24.9 ##
2-APB + 28 d CIH	209.5 ± 7.0	303.7 ± 24.6 ##

*Mean ± SD. Two-way-ANOVA followed by Newman-Keuls tests. ##P > 0.001 vs. initial weight. No differences were found in the initial or final weight between the groups, two way-ANOVA, P = 0.063.*

### 2-Aminoethyl Diphenylborinate Prevented the Increased Right Ventricular Systolic Pressure in Rats Exposed to Intermittent Chronic Hypoxia

**FIGURE 1:**
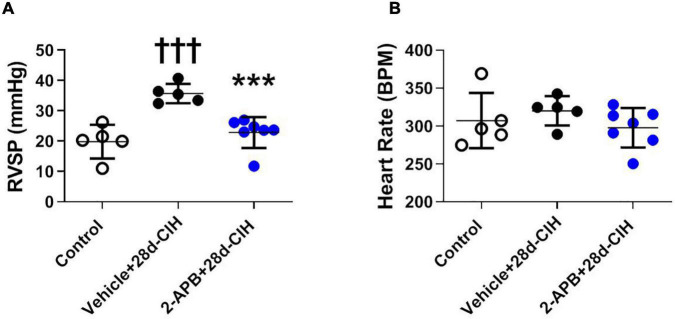
Effect of 2-APB on right ventricular systolic pressure and heart rate in rats exposed to chronic intermittent hypoxia. 2-APB was infused from 14 days of CIH exposure with osmotic pumps at a rate of 10 mg/kg/day. **(A)** Right ventricular systolic pressure (RVSP) (ANOVA *P* = 0.0002). **(B)** Heart rate (HR) (ANOVA *P* = 0.41) in control (empty circles, *n* = 5), Vehicle + 28d-CIH (black circles, *n* = 5), and 2-APB + 28d-CIH rats (blue circles, *n* = 7). Mean ± SD. ^†††^*P* < 0.001 vs. all groups, ***P < 0.001 vs. Vehicle + 28d-CIH. One way ANOVA-followed by Newman Keuls tests.

Following 28 days to CIH, the mean RVSP of the vehicle-treated CIH rats (Vehicle + 28d-CIH) was significantly higher related to the control rats (36.5 ± 3.2 mmHg vs. 19.4 ± 5.5 mmHg, *P* = 0.0001 for Vehicle + 28d-CIH vs. Control rats). Remarkably, 2-APB applied by osmotic pumps from day 14 of CIH (2-APB + 28d-CIH), prevented the increase of RVSP (24.7 ± 5.0 mmHg vs. 36.5 ± 3.2 mmHg, *P* = 0.004 for 2-APB + 28d-CIH vs. Vehicle + 28d-CIH, respectively, see Figure 1A). The heart rate was similar among all groups (Figure 1B).

### 2-Aminoethyl Diphenylborinate Prevented the Pulmonary Vascular Remodeling and the Proliferative Phenotype of PASCM Induced by Intermittent Chronic Hypoxia

**FIGURE 2:**
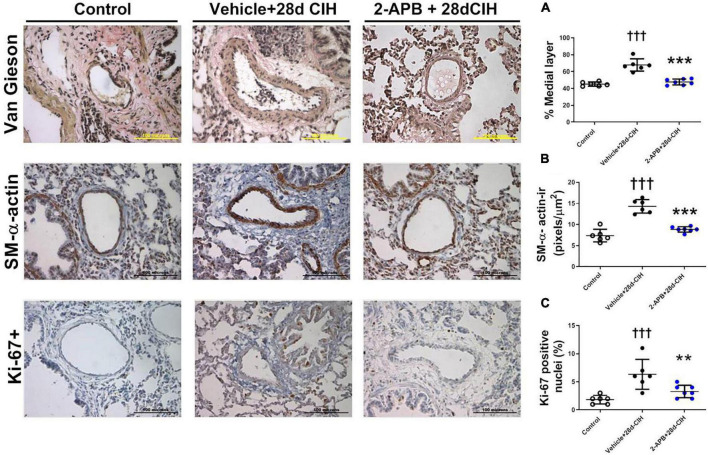
Effect of 2-APB on pulmonary vascular remodeling induced by chronic intermittent hypoxia. **(A)** Medial layer (ANOVA *P* < 0.0001). **(B)** α-actin immunoreactivity in the medial layer (ANOVA *P* < 0.0001). **(C)** Ki-67 positive cells in PASMC (ANOVA *P* = 0.0009) of control (empty circles, *n* = 6), Vehicle + 28d-CIH (black circles, *n* = 6), and 2-APB + 28d-CIH rats (blue circles, *n* = 7). The % of medial layer was measured as (muscle area - lumen area)/(muscle area * 100). Scale bar = 100 μm. ^†††^*P* < 0.001 vs. all groups, **P < 0.01 vs. Vehicle + 28d-CIH ***P < 0.001 vs. Vehicle + 28d-CIH, One way ANOVA-followed by Newman Keuls test.

Exposure to CIH for 28 days increased the thickness of the medial layer of pulmonary arteries (150–300 μm internal diameter) in the vehicle CIH-treated animals related to the control rats (67.4 ± 7.3% vs. 45.2 ± 2.5%, for Vehicle + 28d-CIH and control rats, respectively, *P* < 0.0001). In addition, 2-APB treatment prevented the increase of the medial layer thickness to 47.8 ± 3.5% related to its Vehicle (*P* < 0.0001) (Figure 2A). 2-APB treatment also prevented the increase of α-actin-ir in the medial layer of pulmonary arteries induced by CIH (7.38 ± 1.5 pixels/μm^2^, 14.33 ± 1.6 pixels/μm^2^ and 8.83 ± 0.6 pixels/μm^2^, for Control group, Vehicle + 28d-CIH and 2-APB + 28d-CIH, respectively. *P* < 0.0001) (Figure 2B). Similarly, the percentage of Ki-67 positive cells in the pulmonary medial layer of vehicle-treated rats exposed to CIH was higher in CIH-treated rats than the control rats (6.3 ± 2.7% vs. 1.5 ± 0.8% for Vehicle + 28d-CIH vs. Control group, *respectively*. *P* = 0.0003). 2-APB treatment prevented the increased of PASMC proliferation marker Ki-67 (6.3 ± 2.7% vs. 3.9 ± 1.1% Vehicle + 28d-CIH vs. 2APB-28d-CIH, respectively. *P* = 0.0051) (Figure 2C).

### 2-Aminoethyl Diphenylborinate Prevented the Reduction of the Luminal Vascular Surface Induced by Exposure to Chronic Intermittent Hypoxia

**FIGURE 3:**
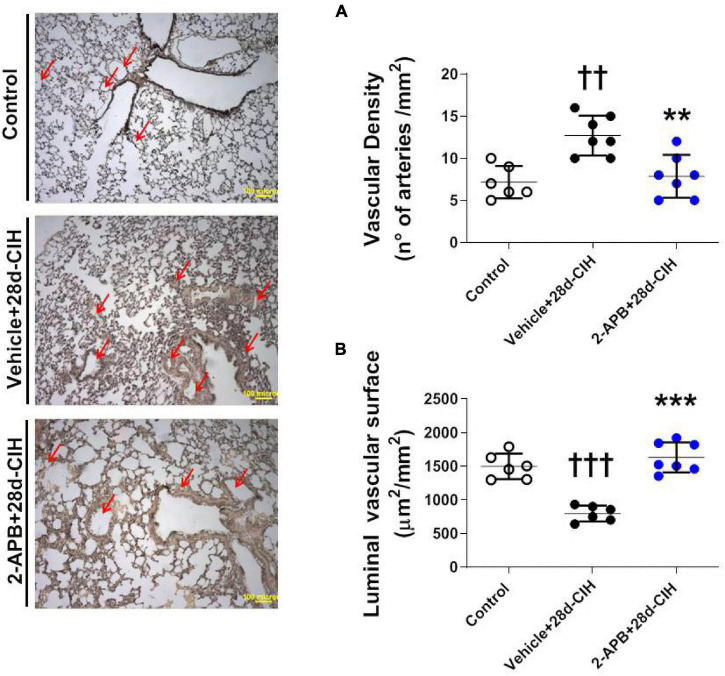
Effect of 2-APB on vascular density and luminal surface in lungs of rats subjected to chronic intermittent hypoxia. Left panel, Reconstruction of 6 lung parenchyma slices stained with Van Gieson. **(A)** Vascular density (ANOVA, *P* = 0.0007). **(B)** Luminal vascular surface (ANOVA, *P* < 0.0001) in controls (empty circles, *n* = 6), Vehicle + 28d-CIH (black circles, *n* = 6), and 2-APB + 28d-CIH rats (blue circles, *n* = 7). Scale bar = 100 μm. Mean ± SD. ^††^*P* < 0.01 vs. all groups, ^†††^*P* < 0.001 vs. all groups, **P < 0.01 vs. Vehicle + 28d-CIH, ***P < 0.001 vs. Vehicle + 28d-CIH. One way ANOVA-followed by Newman Keuls tests.

Exposure to CIH for 28 days increased the number of small pulmonary arteries of 150–300 μm of internal diameter compared to the control rats (12.7 ± 2.3 arteries/mm^2^ vs. 7.2 ± 1.9 arteries/mm^2^, for Vehicle + 28-CIH vs. control rats, respectively. *P* = 0.0005). Treatment with 2-APB normalized the number of arteries (7.9 ± 2.5 arteries/mm^2^ in 2APB + 28-CIH, *P* = 0,001) (Figure 3A). In addition, CIH decreased the vascular luminal surface in the Vehicle-treated animals exposed to CIH as compared to the control group. Nevertheless, 2-APB prevented this decrease despite that rats were exposed to CIH, reaching similar values compared to control group (1497.0 ± 190.9 μm^2^/mm^2^, 797 ± 117.3 μm^2^/mm^2^ and 1632 ± 222.8 μm^2^/mm^2^, for control, Vehicle + 28d-CIH and 2-APB + 28d-CIH, respectively. *P* < 0.0001) (Figure 3B).

### 2-Aminoethyl Diphenylborinate Did Not Prevent the Systemic and Pulmonary Oxidative Stress Induced by Chronic Intermittent Hypoxia

**FIGURE 4:**
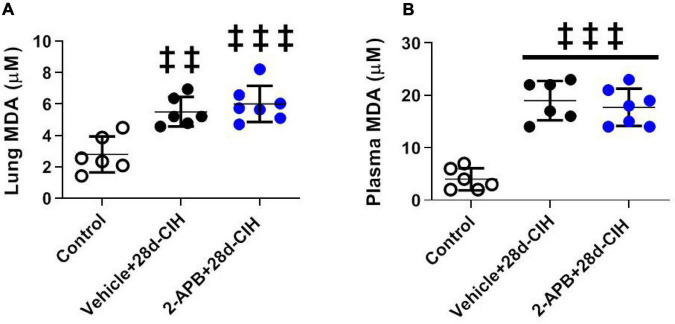
Effect of 2-APB on pulmonary and systemic MDA concentration (TBARS) following chronic intermittent hypoxia. **(A)** MDA lung concentration (ANOVA, *P* = 0.0002). **(B)** Systemic plasma concentration (ANOVA, *P* < 0.0001) in control (empty circles, *n* = 6), exposed to Vehicle + 28d-CIH (black circles, *n* = 6), and 2-APB + 28d-CIH rats (blue circles, *n* = 7). Mean ± SD. ^‡‡^*P* < 0.01 vs. Control, ^‡‡‡^*P* < 0.001 vs. Control. One way ANOVA-Newman followed by Keuls tests.

Exposure to 28 days to CIH increase both systemic and pulmonary MDA concentration in Vehicle-treated rats related to control animals, but 2-APB treatment did not prevent the increase in both the systemic and pulmonary MDA concentration (Figure 4).

### 2-Aminoethyl Diphenylborinate Reduced the Relative Pulmonary Gene Expression of TRPC 1, 4, and 6 but Increased ORAI 1

**FIGURE 5:**
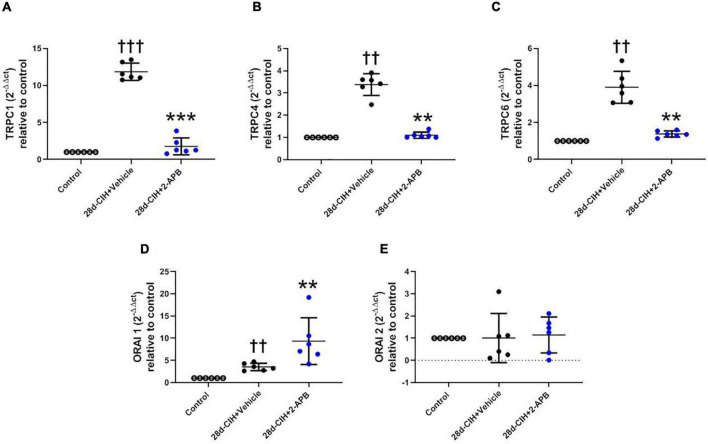
Effect of 2-APB on the relative gene expression of STOC following chronic intermittent hypoxia. **(A)** relative lung gene expression of TRPC1 (ANOVA, *P* = 0.0001). **(B)** TRPC4 (ANOVA, *P* = 0.0017). **(C)** TRPC6 (ANOVA, *P* = 0.0003). **(D)** ORAI1 (ANOVA, *P* = 0.0005), and **(E)** ORAI2 (ANOVA, *P* = 0.9612) in control (empty circles, *n* = 6), Vehicle + 28d-CIH (black circles, *n* = 6), and 2-APB + 28d-CIH rats (blue circles, *n* = 6). Values expressed as 2^–ΔΔCT^ related to the control. Mean ± SD. ^††^*P* < 0.01 vs. all groups, ^†††^*P* < 0.001 vs. all groups **P < 0.01 vs. Vehicle + 28d-CIH, ***P < 0.001 vs. Vehicle + 28d-CIH, One way ANOVA-followed by Newman Keuls tests.

CIH for 28 days increased the relative pulmonary gene expression of TRPC1, TRPC4, TRPC6, and ORAI 1, but not ORAI 2 as compared to control animals (Figure 5). 2-APB-treated rats did not shown an increase in the relative pulmonary gene expression of TRPC 1, TRPC 4 and TRPC6 induced by CIH, being these values similar to control animals (Figures 5A–C). On the contrary, the treatment with 2-APB induced an increase in the relative pulmonary gene expression of ORAI1, which was greater than the one found in the control animals and Vehicle-28d CIH-treated rats (Figure 5D). The expression of ORAI2 remained unchanged among the groups (Figure 5E).

## Discussion

We studied the effects of 2-APB, a drug with inhibitory actions on STOC (Bootman et al., 2002, Castillo-Galán et al., 2016, 2022), in a rat model of CIH-induced pulmonary hypertension (Del Rio et al., 2010, 2012, 2014; Iturriaga et al., 2014; Castillo-Galán et al., 2020). Consistently with previous studies, rats exposed to CIH for 28 days, develop vascular remodeling characterized by an increased thickness of the medial layer in pulmonary arteries and an elevated right ventricular systolic pressure (Nisbet et al., 2009; Jin et al., 2014, 2016; Iturriaga and Castillo-Galán, 2019; Castillo-Galán et al., 2020). The main findings of this study are that 2-APB treatment prevented the raise of RVSP, the pulmonary vascular remodeling characterized by an increased medial layer thickness, the increased α-actin-it and Ki-67 immunoreactive levels, and the lumen vascular surface reduction. In addition, we found that 2-APB did not reduce the lung and plasma oxidative stress, suggesting that the effects of 2-APB on vascular remodeling and pulmonary hypertension are independent of the oxidative stress action. Thus, present results strongly suggest that STOC contribute to the pulmonary hypertension and vascular remodeling in a pre-clinical model of OSA.

Present results showing that 2-APB infusion prevented the increase of RVSP induced by CIH, agree and extended previous studies, which found that 2-APB treatment attenuated the pulmonary vascular remodeling, the increased RVSP and the Fulton index in mice exposed for 4 weeks to sustained hypoxia, as compared to hypoxic animals treated with Vehicle (Smith et al., 2015). Similarly, the daily infusion of 2-APB reduced the vascular remodeling and pulmonary hypertension induced by pregestational hypoxia in newborn lambs in high altitude (Castillo-Galán et al., 2016). Interestingly, our results showed that 2-APB prevented the over-expression of TRPC subunits 1, 4 and 6 induced by CIH in the lung tissue. On the other hand, CIH activates the ET-1 – IP3R pathway in PASMC, releasing Ca^2+^ from the reticulum, which in turn activate STOC (Wang et al., 2012; Castillo-Galán et al., 2020) and raise the intracellular Ca^2+^ concentration (Reyes et al., 2018). In addition, Ca^2+^ activates or stabilized transcription factors that upregulated STOC subunits such as HIFs, NFAT, AP-1, NF-KB and the Platelet Derived Growth Factor (Shibasaki et al., 1996; Timmerman et al., 1996; Dolmetsch et al., 1997; Chen et al., 1998; Salnikow et al., 2002; Mottet et al., 2003; Ogawa et al., 2012; Jiang et al., 2014; Domínguez-Rodríguez et al., 2015; Wang et al., 2015, 2017). Taken together, the evidence suggests that 2-APB would induce a decrease in intracellular calcium (Parrau et al., 2013), which may inhibit the upregulation of TRPC1, TRPC4 and TRPC6. However, more studies are required to determine if all of these factors that regulated STOC changed in the lung tissue following CIH.

In this study, 2-APB was administered with osmotic pumps at a delivery rate of 10 mg/kg per day, which is similar to the daily dose range used by Smith et al. (2015), Castillo-Galán et al. (2016, 2022) and Jain et al. (2021) to block STOC *in vivo*. Indeed, Smith et al. (2015) treated chronic hypoxic mice with 2-APB (1 mg/kg/day i.p.) to reduce pulmonary hypertension and right ventricular enlargement. Castillo-Galán et al. (2016, 2022), administered 2-APB (10 mg/kg/day i.v.) to neonatal lambs with a partial or full gestation in high altitude to ameliorate the pulmonary hypertension and vascular remodeling, and Jain et al. (2021) administrated 2-APB (1 mg/kg per day i.p.) to attenuate pulmonary hypertension in hypoxic mice. We did not measure the plasma concentrations of 2-APB after its administration, but Parrau et al. (2013) estimated that a single dose of 2-APB (8 mg/kg i.v.) would reach a concentration in the extracellular space in the range of 10–100 μM, assuming a distribution volume of 40% of the body weight. The same order of 2-APB concentration blocks STOC in isolated pulmonary arteries from hypoxic lambs (Parrau et al., 2013) and in HEK293 cells transfected with Orai/Stim or TRPC subunits (Lievremont et al., 2005; Peinelt et al., 2008).

Since 2-APB is a non-selective STOC blocker, we cannot preclude effects of 2-APB at the IP3R level. This make it uncertain to determine to what extent the effects of 2-APB on pulmonary vascular remodeling and hypertension are due to its effects on any of these targets. The participation of IP3R on the response of pulmonary arteries to hypoxia is controversial and depend on the species and type of vessel studied. While in rat distal pulmonary artery PASMC, both Ca^2+^ release from intracellular stores and Ca^2+^ entry from the extracellular space induced by hypoxia are abolished by IP3R blockade with xestonpongin-C (Wang et al., 2012), in canine pulmonary artery PASMC, Ca^2+^ entry mediated by STOC in response to hypoxia is unaffected by IP3R blockade (Ng et al., 2008). On the other hand, in rat main pulmonary arteries, both cytosolic Ca^2+^ increase and contraction in response to hypoxia are biphasic, with a rapid transient fast response followed by a slowly developing and sustained response. In these arteries, the contraction in response to hypoxia develop without changes in IP3 content (Jin et al., 1993), Indeed, it has been proposed that STOC account for the sustained Ca^2+^ increase in response to hypoxia (Sylvester et al., 2012). Moreover, in isolated pulmonary arteries of newborn lambs, the relaxation elicited by SKF-96365, a specific STOC blocker without effect on IP3R is similar to the relaxation induced by 2-APB, and both of them increased in pulmonary arteries from hypoxic lambs (Parrau et al., 2013). Taken together these observations suggest that 2-APB may act on IP3R and STOC, but the final effect is an indirect or a direct reduction of Ca^2+^ entry mediated by STOC in pulmonary PASMC from animals exposed to hypoxia. Considering that 2-APB is not a selective STOC blocker, new drugs with a selective effects on ORAI1/STIM1 interaction are needed to evaluate the role of the STOC in OSA patients with pulmonary hypertension (Reyes et al., 2018; Waldron et al., 2019).

Chronic intermittent hypoxia induces oxidative stress at the pulmonary and systemic level, which may affect calcium homeostasis and contribute to the lung vascular remodeling (Del Rio et al., 2010, 2012; Jin et al., 2014, 2016; Iturriaga and Castillo-Galán, 2019; Castillo-Galán et al., 2020). Antioxidants such as procyanidin and melatonin reduced the elevated RVSP and the vascular remodeling induced by CIH (Jin et al., 2014, 2016). The activation of TRPC1 and TRPC6 by the transcription factor BMP4 in rat PASMC depended on the increased ROS levels mediated by NOX4 (Jiang et al., 2014). In addition, it has been proposed that the increase in ROS levels induced by hypoxia, activates the ryanodine receptors (RyR), which in turn induces the release of the reticular Ca^2+^, activating STIM1, and allowing the interaction with ORAI in the plasma membrane and subsequent activation of the STOC (Suresh and Shimoda, 2016). These results suggest that the effects of antioxidants may be related with the inactivation of STOC and subsequently decrease in intracellular calcium. Although an antioxidant action of 2-APB has been shown in ischemia/reperfusion damage in mice and rat cardiomyocytes (Morihara et al., 2017; Shen et al., 2021), our results showed that 2-APB did not prevent the systemic and lung oxidative stress, suggesting that the effects of 2-APB on PASMC in the lung are not related by a reduction in oxidative stress. Thus, it is likely that the effects of 2-APB were related to its inhibitory action on the increased intracellular Ca^2+^ concentrations induced by STOC (Bootman et al., 2002). Thus, the treatment of hypoxic pulmonary hypertensive rats with 2-APB, has a strong effect preventing the increase of RVSP and the pulmonary vascular remodeling without any significant action on the oxidative stress markers.

## Conclusion

Present results indicated that 2-APB treatment to rats exposed to CIH prevented the increased RVPS and the pulmonary vascular remodeling, without effects on both systemic and pulmonary oxidative stress markers, suggest that STOC are and attractive therapeutic target to prevent pulmonary hypertension in OSA. In summary, present results showed that 2-APB strongly ameliorated the pulmonary vascular alterations induced by CIH, indicates that the STOC inhibition may potentially be used to treat the pulmonary hypertension induced by OSA.

## Data Availability Statement

The raw data supporting the conclusions of this article will be made available by the authors, without undue reservation.

## Ethics Statement

The animal study was reviewed and approved by Scientific Ethical Committee for the Animal and Environment Care Pontificia Universidad Católica de Chile, Santiago, Chile (ID:180803006).

## Author Contributions

SC-G and BR performed the experiments. SC-G and RI contributed to the conception and design of research, analyzed the data, interpreted the results, and drafted the manuscript. All authors revised the manuscript and approved final version of the manuscript.

## Conflict of Interest

The authors declare that the research was conducted in the absence of any commercial or financial relationships that could be construed as a potential conflict of interest.

## Publisher’s Note

All claims expressed in this article are solely those of the authors and do not necessarily represent those of their affiliated organizations, or those of the publisher, the editors and the reviewers. Any product that may be evaluated in this article, or claim that may be made by its manufacturer, is not guaranteed or endorsed by the publisher.
